# A Mixture Method for Robust Detection HCV Early Diagnosis Biomarker with ML Approach and Molecular Docking

**DOI:** 10.3390/ijms24087207

**Published:** 2023-04-13

**Authors:** Maryam Gholizadeh, Joanna Łapczuk-Romańska, Mariola Post, Nina Komaniecka, Seyed Reza Mazlooman, Lars Kaderali, Marek Droździk

**Affiliations:** 1 Institute for Bioinformatics, University Medicine Greifswald, 17475 Greifswald, Germany; 2 Department of Experimental and Clinical Pharmacology, Pomeranian Medical University, 70-111 Szczecin, Poland; 3 Department of General and Transplantation Surgery, County Hospital, 71-455 Szczecin, Poland; 4 Department of Psychiatry and Psychotherapy, University Medicine Greifswald, 17475 Greifswald, Germany

**Keywords:** HCV diagnosis biomarker, machine learning algorithms, molecular docking, therapeutic target

## Abstract

Given the substantial correlation between early diagnosis and prolonged patient survival in HCV patients, it is vital to identify a reliable and accessible biomarker. The purpose of this research was to identify accurate miRNA biomarkers to aid in the early diagnosis of HCV and to identify key target genes for anti-hepatic fibrosis therapeutics. The expression of 188 miRNAs in 42 HCV liver patients with different functional states and 23 normal livers were determined using RT-qPCR. After screening out differentially expressed miRNA (DEmiRNAs), the target genes were predicted. To validate target genes, an HCV microarray dataset was subjected to five machine learning algorithms (Random Forest, Adaboost, Bagging, Boosting, XGBoost) and then, based on the best model, importance features were selected. After identification of hub target genes, to evaluate the potency of compounds that might hit key hub target genes, molecular docking was performed. According to our data, eight DEmiRNAs are associated with early stage and eight DEmiRNAs are linked to a deterioration in liver function and an increase in HCV severity. In the validation phase of target genes, model evaluation revealed that XGBoost (AUC = 0.978) outperformed the other machine learning algorithms. The results of the maximal clique centrality algorithm determined that CDK1 is a hub target gene, which can be hinted at by hsa-miR-335, hsa-miR-140, hsa-miR-152, and hsa-miR-195. Because viral proteins boost CDK1 activation for cell mitosis, pharmacological inhibition may have anti-HCV therapeutic promise. The strong affinity binding of paeoniflorin (−6.32 kcal/mol) and diosmin (−6.01 kcal/mol) with CDK1 was demonstrated by molecular docking, which may result in attractive anti-HCV compounds. The findings of this study may provide significant evidence, in the context of the miRNA biomarkers, for early-stage HCV diagnosis. In addition, recognized hub target genes and small molecules with high binding affinity may constitute a novel set of therapeutic targets for HCV.

## 1. Introduction

Hepatitis C is a multifactorial disease, with a reported global prevalence of 56.8 million hepatitis C virus (HCV) infections on 1 January 2020 (95% uncertainty interval (UI) 55.2–67.8). Although this number is lower than in 2015, forecasts based on studies within 235 nations and territories indicate that we are not on track to meet global elimination targets by 2030 due to COVID-19 [1]. Recently developed direct-acting antiviral agents (DAAs) targeting viral NS3 protease and NS5A and NS5B polymerases are highly effective in curing patients with HCV. However, global eradication of HCV remains complicated due to the lack of a HCV vaccine, the possibility of drug-resistant mutations, progression of severe liver disease in DAA-cured individuals, and other newly emerging problems. The pathogenic mechanisms that sustain the liver damage in HCV infection may underlie even other liver diseases, thus potentially becoming new effective early diagnostic markers and/or therapeutic targets, even in non-infected patients [2]. Notably, genome expression alteration occurs prior to histologic evidence of liver disease progression, suggesting that events that develop during the acute phase of infection influence patients outcome. Further, histological assessments may be biased, and interpretations of hepatic structural abnormalities vary among pathologists [3]. Therefore, it is crucial to identify accurate biomarkers that can detect liver damage early and precisely. Despite dysregulated host gene expression, there is growing evidence that microRNAs (miRNAs) play a crucial role in the biological interaction between HCV and the host cell, which might be used by host cells to control viral infection [4,5]. All those studies reveal the indispensable role of miRNAs in viral infection and disease progression. MicroRNAs are small, non-coding RNAs that complement each other non-precisely and bind to the 3′ untranslated regions of target mRNA, resulting in mRNA dysregulation. Increasing evidence demonstrates that miRNAs are one of the central factors in the interaction network between virus and host. However, the expression of miRNAs for disease treatment has so far been a difficult task due to their variable nature in different situations [6], but the same nature of being differentially expressed under different conditions allows miRNAs to be used as biomarkers for diseases. Some special miRNAs are highly correlated with the progression of liver-specific pathologies, and altered levels of miRNAs are even more sensitive and specific than those of conventional proteins. Some of them can therefore serve as novel, less invasive diagnostic and prognostic biomarkers for HCV-infected patients with liver diseases. In addition, they are promising therapeutic targets for the development of new anti-HCV agents. 

The first aim of the present study was to assess whether the expression of miRNAs correlated with different functional states of HCV in order to identify a non-invasive diagnostic biomarker. Therefore, the expression of 188 miRNAs in 42 HCV livers at different functional states and 23 normal livers was determined using RT-qPCR. These data were further applied to screen out differentially expressed miRNAs (DEmiRNAs) in HCV and normal liver tissues. Second, to discern the key genes that may serve as therapeutic targets to hit with drugs, the potential target genes of DEmiRNA were predicted using miRNet. To further validate target genes, we employed the GEO database to download GSE34798—the HCV mRNA expression dataset. Then, five ensemble machine learning algorithms (Random Forest, Adaboost, Bagging, Boosting, and XGBoost) were conducted on the dataset. After evaluation of the models, important features were selected based on the best model, and validated target genes were screened using Venny plot. To find the hub target genes, after acquiring the PPI network, the hub target genes were screened using MCODE (Cytoscape plug-in Molecular Complex Detection) and the cytoHubba plug-in. To explore compounds that might hit the hub target gene, the structure of 18 small molecules with anti-hepatic fibrosis action was downloaded, and molecular auto-docking was performed using AutoDock Tool 1.5.6.

## 2. Results

### 2.1. Differentially Expressed miRNA

**Figure 1:**
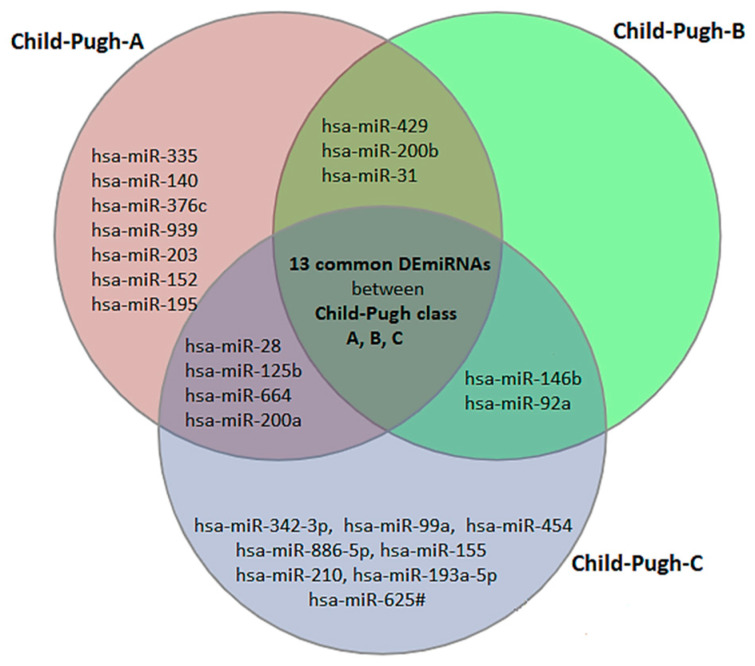
The Venn diagram illustrates that 13 differentially expressed miRNAs (DEmiRNAs) are shared by Child–Pugh classes A, B, and C, and could be used as biomarkers for HCV diagnosis but not disease stage. However, the diagram also depicted 8 specific biomarkers for early diagnosis of HCV, including hsa-miR-335, hsa-miR-140, hsa-miR-376c, hsa-miR-939, and 8 DEmiRNAs, which can all be potential biomarkers for functional stage C.

The result of ANOVA indicated that the expression of 34 miRNAs was significantly different in HCV patients compared to normal (8 upregulated and 26 downregulated). When comparing normal livers to Child–Pugh class A, B, and C, we identified 28 (6 upregulated and 22 downregulated), 19 (5 upregulated and 14 downregulated) and 28 (6 upregulated and 22 downregulated) differentially expressed miRNAs, respectively (Supplementary File S1). The result of theVenn diagram shows that 13 DEmiRNAs are common between Child–Pugh class A, B, and C and 8 DEmiRNAs only in Child–Pugh class A but not in child- B or C, which means that these miRNAs can be potential biomarkers for early diagnosis, because their expression has changed specifically in the patients of functional stage A. Moreover, 8 DEmiRNAs were only in Child–Pugh class C but not in Child-A or B (potential biomarkers for stage C). Our result did not show any particular DEmiRNAs only in Child–Pugh class B, which means that the exact differentiation in moderate state (Child-B) can be difficult based on miRNAs biomarkers (Figure 1 and Supplementary File S2). Our results of the statistical analysis further show that the expression of hsa-miR-342-3p, hsa-miR-886-5p, and hsa-miR-210 from healthy situation to functional stage A and B slowly increased (not significantly), but at functional stage C it significantly increased (Supplementary File S3). 

### 2.2. Validated Target Genes Using Ensemble Machine Learning Algorithms 

**Figure 2:**
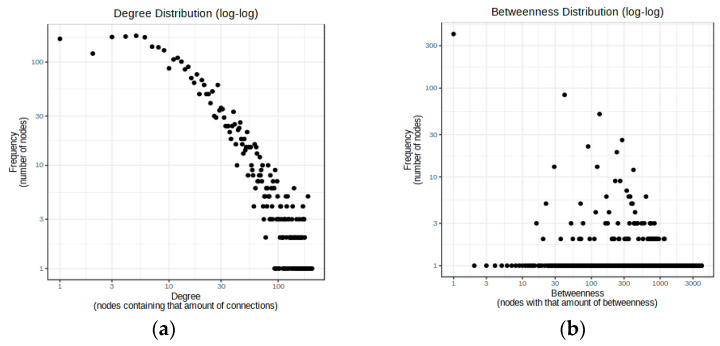
(**a**) Degree distribution graph for down-regulated specific DEmiRNAs in Child–Pugh A and predicted target genes. The diameter of the network is 4, the average path length is 2.26, and the nodes of hsa-mir-152 (158) and hsa-mir-195 (119) have the greatest number of connections; (**b**) Betweenness distribution graph for down-regulated specific DEmiRNAs in Child–Pugh C and predicted target genes. The diameter of the network is 5, the average path length is 2.01, and hsa-mir-155 (0.907471357) and hsa-miR-99a (0.6527755) have higher betweenness centrality and the greatest number of shortest paths, which indicates the key role of these nodes in the network.

Generally, miRNAs perform posttranscriptional functions by base-pairing to the mRNA 3′ untranslated regions. Therefore, the miRNet database was applied to predict the target genes of up-regulated and down-regulated specific DEmiRNAs in functional stages A and C, respectively (Supplementary File S4). Table 1 and Figure 2 represent features of the network between DEmiRNAs and predicted target genes. Topological analysis of the networks shows that hsa-mir-152 and hsa-mir-195 have the greatest number of connections between down-regulated specific DEmiRNAs in Child–Pugh A. Moreover, in the network of down-regulated specific DEmiRNAs in Child–Pugh C, hsa-mir-155 and hsa-miR-99a have higher betweenness centrality, and in the network of up-regulated DEmiRNAs, hsa-miR-886-5p and hsa-miR-342-3p play key roles. To validate the predicted target genes, five ensemble machine learning algorithms were applied to 22,149 genes from 459 samples with HCV and 459 samples without HCV obtained from the GSE34798 data set.

The predictive performance of five algorithms for classifying genes as DEGs or non-DEGs was evaluated. Positive predictive value (PPV), recall (sensitivity), F-score (a harmonic mean of sensitivity), precision, AUC, and Brier score (BS) were used to evaluate the models. The results of the predictive performance comparison models are displayed in Table 2. With an accuracy of 0.978, the XGBoost model demonstrated superior performance across all evaluation criteria compared to the other machine learning algorithms.

**Figure 3:**
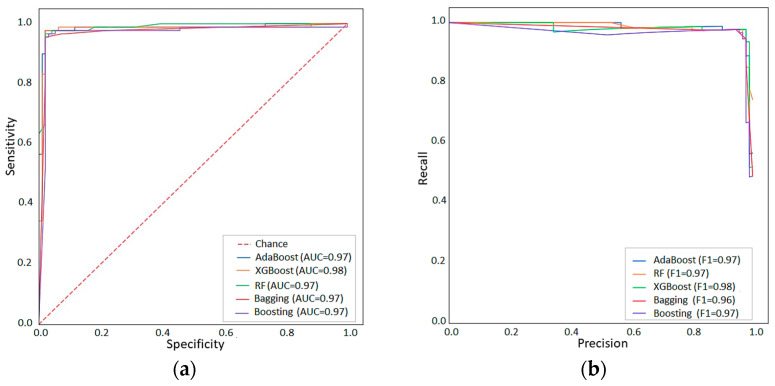
Model performances for XGBoost, AdaBoost, Boosting, Bagging, and Random Forest are shown as ROC-AUC (**a**) and Precision-Recall Curves (**b**).

To assess the performance of the models on test sets, the AUC-ROC for each model was calculated. The XGBoost has the best performance, with an AUC over 0.97 in the test set (Figure 3a). The recall and PPV of the machine learning algorithms were also high, over 0.96. The precision–recall curve is represented in Figure 3b. In this curve, the XGBoost model shows the best performance with a higher value (0.98).

Next, XGBoost was chosen to extract the top features (important DEGs) that were subjected to recursive feature elimination. Through recursive feature elimination, the least number of features were obtained (Supplementary File S5). Based on XGB feature importance analysis, 970 DEGs were detected (620 up-regulated, 350 down-regulated). Previous studies have shown a negative feedback relationship between miRNA and mRNA. Thus, a Venn diagram was utilized to obtain the intersection between down-regulated (up-regulated) DEGs and target genes of up-regulated (down-regulated) DEmiRNAs (Supplementary File S6):

- Target genes of down-regulated DEmiRNAs in Child–Pugh A (3722) vs. up-regulated DEGs (620) = 148 up-regulated genes
- Target genes of up-regulated DEmiRNAs in Child–Pugh A (786) vs. down-regulated DEGs (350) = 52 down-regulated genes
- Target genes of down-regulated DEmiRNAs in Child–Pugh C (4724) vs. up-regulated DEGs (620) = 195 up-regulated genes
- Target genes of up-regulated DEmiRNAs in Child–Pugh C (240) vs. down-regulated DEGs (350) = 37 down-regulated genes

### 2.3. Screen Hub Target Genes

**Figure 4:**
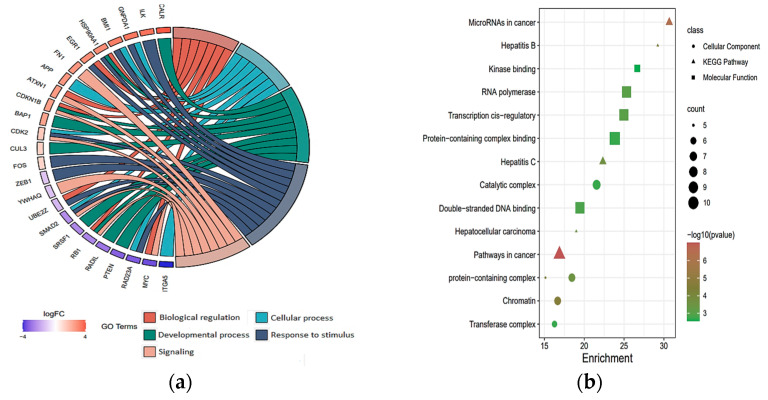
Gene ontology analysis of key target genes; (**a**) the chord plots illustrate the relationship between significant GO terms and target genes, and most of the target genes involved in biological processes of response to stimulus; (**b**) The scatter plot shows cellular component, molecular function, and KEGG enrichment analysis.

To further investigate the hub target genes, a PPI network for target genes in each category was constructed using protein interaction data obtained from the STRING (Search Tool for the Retrieval of Interacting Genes) database and then visualized using Cytoscape (Supplementary File S7). A statistical summary of the networks is presented in Supplementary File S8. For the target genes of down-regulated DEmiRNAs in Child–Pugh A and C networks, we first employed the MCODE (Molecular Complex Detection) plugin from Cytoscape to find the top clusters derived from them (Supplementary File S9), and we then employed the cytoHubba to identify the hub genes from the top clusters using the maximal clique centrality (MCC) algorithm, and the genes with the top MCC values were considered hub genes. Due to the small size of the networks of down-regulated target genes (Child–Pugh A and C), cytoHubba was directly applied to identify the top target genes. For down-regulated DEmiRNAs in Child–Pugh A, the following target genes were identified: STAT1, TGFBR1, PTEN, CUL3, FOS, BAP1, SLC12A4, GNPDA1, CDK1, for up-regulated DEmiRNAs in Child–Pugh A: SMAD4, MELK, SRSF1, for down-regulated DEmiRNAs in Child–Pugh C: ATXN1, CDKN1B, EGR1, RB1, CALR, FN1, UBE2Z, YWHAQ, ZEB1, ITGA5, and for up-regulated DEmiRNAs in Child–Pugh C: MYC, ILK, GTF2A1, CDK2. The results of the gene ontology enrichment analysis indicate that the majority of the most abundant genes were enriched in biological regulation, cellular processes, developmental processes, metabolic processes, and responses to stimulus signaling (Figure 4a). Moreover, these genes were mainly involved in pathways such as cancer, hepatocellular carcinoma, hepatitis C, hepatitis B, and microRNAs in cancer and play key roles in some molecular functions, including double-stranded DNA binding, protein-containing complex binding, transcription cis-regulatory, RNA polymerase, and kinase binding (Figure 4b).

### 2.4. Binding Affinity between Target Gene and Anti-Hepatic Fibrosis Small Molecules

**Figure 5:**
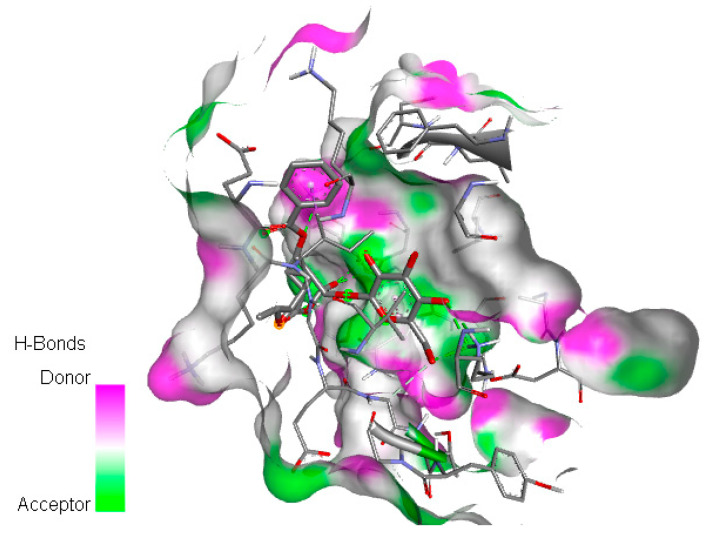
Docking of paeoniflorin molecule on CDK1 protein with six hydrogen bond acceptors, two hydrophobic bonds (Pi-Sigma and Pi-Alkyl), and binding site is visualized through its surface.

After removing water and ligand from CDK1 (6GU6, pdb resolution 2.33 Å), polar hydrogens were added using the Discovery Studio Visualizer tool. Ligand preparation and docking between protein and ligands were performed using Autodock Vina (with no change in rotatable bonds and active torsion for the ligand). All docked poses had a root mean square deviation (RMSD) value below 2.0 Å. The result of the united atom scoring function shows the highest binding affinities between CDK1 and paeoniflorin (−6.32 kcal/mol) and diosmin (−6.01 kcal/mol). Figure 5 illustrates the visualization of the docking of the paeoniflorin molecule on the CDK1 protein. Moreover, detailed information for all 18 small molecules, including binding energy between CDK1 and natural small molecules, chemical formula, and mechanisms, is provided in Table 3.

## 3. Discussion

More than half of HCV infected patients develop chronic infection. Therefore, early detection is essential for preventing or delaying the disease progression [7]. miRNAs are closely correlated with liver-specific disease progression, and the altered levels of miRNAs have even higher sensitivity and specificity than proteins. Therefore, some of them can serve as novel diagnostic biomarkers in HCV-infected patients. Thus, in the current study, 188 miRNA expressions in HCV patients and normal livers was compared to find DEmiRNAs as biomarkers in different functional states of HCV (A, B, and C). Our results show that in liver tissues of HCV patients in the early stage, the expression of hsa-miR-335, hsa-miR-939, hsa-miR-140, hsa-miR-376c, hsa-miR-203, hsa-miR-152, and hsa-miR-195 is significantly decreased, whereas the expression of hsa-miR-27b is highly up-regulated compared to the samples without liver disease, which can be potential biomarkers for the diagnosis of HCV in the early stage. The miR-27b is involved in lipid regulatory pathways and plays a crucial role in a self-inhibiting mechanism in HCV by downregulating the genes engaged in lipid metabolism that are required for HCV replication [8]. Therefore, an increase in miR-27b expression may serve as a potential marker for early-stage HCV patients. Although some studies demonstrated that miR-335, hsa-miR-203, and hsa-miR-152 can serve as biomarkers for the early diagnosis of HCV, but it is still debated [9,10,11]. Then, to discern the target genes that may serve as a therapeutic target in the early stage of HCV, the potential of DEmiRNA target genes, using miRNet were predicted.

To further verify the target genes, the first five ensemble machine learning algorithms (Random Forest, Adaboost, Bagging, Boosting, and XGBoost) were conducted on 22,149 genes of 459 samples with HCV, and 459 samples without HCV to find the best model. Evaluation of models based on PPV, recall, F-score, accuracy, AUC, and BS indicated that the XGBoost (AUC 0.978) model presented better performance in all evaluation metrics than the other machine learning algorithms. The result of the intersection between up-regulated DEGs (output of feature selection based on XGBoost) and target genes of down-regulated DEmiRNAs in Child–Pugh A shows 148 validated up-regulated target genes. To find hub target genes, after acquiring the PPI network, the hub target genes were screened using MCODE and the cytoHubba plug-in. The results of the maximal clique centrality algorithm indicate SLC12A4 (target genes for hsa-miR-939) and CDK1 (target genes for hsa-miR-335, hsa-miR-140, hsa-miR-152, and hsa-miR-195) as key up-regulated target genes in the early stage of HCV disease.

This transporter-related gene has been reported to be differentially expressed in HCV-infected patients by a number of prior studies [12,13,14]. Solute carrier family 12 member 4 (SLC12A4) is one of the essential genes for HCV RNA replication; therefore, suppression of this gene may result in inhibition of HCV replication. CDK1 is a key regulatory kinase of the cell cycle in the CDK family. A previous study demonstrated that CDK1 is up-regulated in HCV [15] and the viral protein increases the activity of the cyclin B1-CDK1 complex through the MAPK p38 and JNK pathways [16]. As CDK1 activation is required for common regulatory processes of the cell cycle, inhibition or interference by drugs has the potential to be an effective method of HCV treatment and progression prevention. To assess the potency of compounds that might hit CDK1, molecular auto-docking was performed. The observed binding energy of natural compounds artesunate and betulinic acid for CDK1 indicates their promising anti-fibrotic effects. Likewise, peoniflorin (from *Paeonia lactiflora*) targeting CDK1 demonstrates various effects on liver diseases. Investigation in clinical trials shows that this small molecule plays a key role in inhibiting liver inflammation through regulating multiple signaling pathways. Diosmin is a natural flavone that is proved to promote vascular health, but only part of the experimental results indicates its anti-inflammatory and antioxidant effects, which might be related to CDK1 activity.

## 4. Materials and Methods

### 4.1. Patient Characteristics and Specimens

RT-qPCR was used to determine the expression of 188 miRNAs in the livers of 42 HCV patients with different functional states (Child–Pugh class B (*n* = 11) and C (*n* = 7), as well as Child–Pugh class A (*n* = 23), and 23 normal livers (control). Normal liver tissue samples were obtained from patients without liver disease, undergoing resection of metastases from colon cancer at a distance of at least 5 cm from the tumor site. Histological examination verified the non-existence of pathological indicators in the collected tissues (the samples were used as controls in the previously published study). During elective liver transplantation, liver parenchymal tissue samples from patients with Hepatitis C infection (as determined by the standard clinical criteria) were obtained. The stage of liver dysfunction was categorized using the Child–Pugh score. The characteristics of the subjects are presented in Table 4. Tissue biopsies were taken from livers (control and pathological) under standard general anesthesia no later than 15 min after blood flow arrest. The liver samples were immediately snap frozen in liquid nitrogen for protein analysis or immersed in RNAlater (Applied Biosystems, Darmstadt, Germany) for RNA analysis, and then stored at −80 °C. The study protocol was approved by the Bioethics Committee of the Pomeranian Medical University.

### 4.2. Micro-Ribonucleic Acids Expression and Statistical Analysis

From 40–50 mg of tissue samples, total RNA (including small RNA) was isolated using the Direct-zol RNA Miniprep Plus Kit (Zymo Research, Irvine, CA, USA); RNA concentration was then measured using a NanoDrop ultraviolet (UV) spectrophotometer (Thermo Fisher Scientific, Waltham, MA, USA). Reverse transcription was performed using a TaqMan MicroRNA Reverse Transcription Kit (Applied Biosystems, Darmstadt, Germany) in two separate reactions, each containing a different pool of Megaplex RT Primers (Human Pools A and B; Applied Biosystems, Darmstadt, Germany) and 500 ng of total RNA in a reaction volume of 7.5 µl. Finally, quantitative PCR was performed using the TaqMan Array Cards (TaqMan Array Human MicroRNA A + B Cards Set v3.0, Thermo Fisher Scientific, Waltham, MA, USA) in a ViiA7 Real-Time PCR system (Thermo Fisher Scientific, Waltham, MA, USA). Of the 754 analyzed miRNAs, 188 unique miRNAs that had a Ct value below 32 were selected for further analysis (as recommended by protocol from the assay provider). The relative quantity (RQ) of each miRNAs was calculated using the ΔCt method in relation to the mean expression of three endogenous controls (stably expressed small noncoding RNAs: U6 snRNA, RNU44, and RNU48). To investigate differentially expressed microRNAs (DEmiRNAs) between disease groups (in total and separately in different functional states) and control groups, an ANOVA was performed using R version 4.1.3 (http://www.Rproject.org, accessed on 20 December 2022) on the number of normalized microRNA counts. The Holm–Bonferroni method was used to correct for multiple testing, and miRNAs with an adjusted *p*-value ≤ 0.05 were considered DEmiRNAs.

### 4.3. Prediction and Validation of Potential Target Genes

Based on liver tissue, the miRNet database (https://www.mirnet.ca/miRNet/home.xhtml, accessed on 14 January 2023) was used to predict the target genes of the differentially expressed miRNAs. Key DEmiRNAs were extracted based on a topological analysis of miRNA-target gene networks. To further validate the screened target genes for DEmiRNAs, we employed the GEO database to download the HCV mRNA expression datasets. We predicted DEmiRNAs between 22,149 genes of patients with HCV and subjects without HCV using the GSE34798 dataset to further verify the target genes.

### 4.4. Selection of the Best Classification Model and Validation Based on Machine Learning Algorithms

To validate predicted target genes, microarray data were subjected to five of the most frequently recommended machine learning models from prior research. In order to prevent data overfitting, ensemble methods with high detection power were used to build stable models for predicting significant genes. At this point, ensemble methods including XGBoost, AdaBoost, Boosting, Bagging, and Random Forest were used to extract effective genes in hepatitis C disease from gene expression data. To adjust the hyper parameters, the random selection algorithm with ten-fold cross-validation was used, as suggested by Bargstra and Bengio [17]. All the data analyses were carried out in the Python programming language (v. 3.8) with the scikit-learn library. Results were visualized in Matplotlib (v. 3.1.328, author: John D. Hunter) and Seaborn (v. 0.10.0, author: Michael Waskom). To assess the performance of the models, well-known measures such as accuracy, precision, or positive predictive value (PPV), recall (sensitivity), F-Score (a harmonic mean of sensitivity), AUC, Brier score (BS), and Matthews correlation coefficient (MCC) were analyzed. These criteria were obtained using the reports of each classifier’s learning algorithm and the confusion matrix. Table 5a represents the confusion matrix and contains four categories when the predictive scores are binary. The false-positive component (FP) represents the number of DEGs incorrectly placed in the non-DEG category by the model. True positive (TP) indicates the number of DEGs placed in this category by the model. False-negative (FN) means the number of non-DEGs incorrectly placed in the DEGs category by the model. True negative (TN) indicates the number of non-DEGs correctly placed in this category by the model. The formula for the evaluation metric to assess the performance of the models is represented in Table 5b.

The BS was also utilized to evaluate the performance of the models. This criterion evaluates the overall accuracy of the model, which is represented by the square of the difference between the actual value and the predicted value; a smaller value is preferable. MCC takes values between -1 and 1, and a high value means that both classes (DEGS and non-DEGs) are predicted accurately. Furthermore, ROC (receiver operating characteristic) and precision-recall plots were used to select the best model. The model that generated the highest values for all metrics was chosen to extract the top features, which were then subjected to recursive feature elimination. Subsequently, to find the boost potential target genes and due to the negative feedback relationship between miRNA and mRNA, we employed Venny 2.1.0 (https://bioinfogp.cnb.csic.es/tools/venny/, accessed on 25 January 2023) to intersect down-regulated (up-regulated) DEGs with target genes of screened up-regulated (down-regulated) DEmiRNAs to obtain the boost potential target genes.

### 4.5. Construction and Topological Analysis Target Gene Networks

PPI networks were constructed using the Search Tool for the Retrieval of Interacting Genes (STRING) database (http://string-db.org, accessed on 3 February 2023) with the highest confidence threshold (0.900) to interpret the interactive relationships between validated target genes. Each score is derived by benchmarking analysis and generally corresponds to an estimate of the likelihood that a given association describes a functional connection between two genes. Using the Network Analyzer plug-in, topological properties were calculated for each node of the constructed networks in order to identify key target genes. Top modules were screened using the Cytoscape plug-in Molecular Complex Detection (MCODE, http://apps.cytoscape.org/apps/mcode, accessed on 6 February 2023). The following parameters were set for the Cytoscape analysis: degree cut-off = 2, node score cut-off = 0.2, k-core = 2, and maximum depth = 100. Next, the cytoHubba plug-in was utilized to identify the hub genes. 

### 4.6. Functional and Pathway Enrichment Analysis

To figure out the potential functional role of key target genes, GO annotation and KEGG pathway enrichment analyses were performed for each miRNA using its respective target genes. Visualization of data was performed using the igraph package in R.

### 4.7. Functional and Pathway Enrichment Analysis

To explore compounds that might bind to a significant target gene, we downloaded the structures of 18 small molecules with anti-hepatic fibrosis action in pdb format from the PubChem database (https://pubchem.ncbi.nlm.nih.gov/, accessed on 7 January 2023), as well as the crystal structure of CDK1 (6GU6) from the RCSB PDB (https://www1.rcsb.org/, accessed on 7 January 2023). Small molecules that are compounds from natural products and are from four different classes include alkaloids, flavonoids, terpenes, and phenols. Molecular docking was performed using the Discovery Studio Visualizer tool and Auto Dock Tools 1.5.6 and a rigid docking protocol that used a genetic algorithm to generate binding poses of the protein–ligand complexes.

## 5. Conclusions

For the treatment and prevention of HCV, resistance to DAA and impediments to the development of a vaccine continue to pose the major challenges. Here, robust potential biomarkers to aid in the early diagnosis of HCV have been identified, along with potential target genes and anti-hepatic fibrosis molecules for HCV therapy. Altogether, these data support the idea that an alteration in hsa-miR-27b, hsa-miR-335, hsa-miR-140, hsa-miR-376c, hsa-miR-939, hsa-miR-203, hsa-miR-152, and hsa-miR-195 is associated with HCV in the early stage and hsa-miR-342-3p, hsa-miR-99a, hsa-miR-454, hsa-miR-886-5p, hsa-miR-155, hsa-miR-210, and hsa-miR-193a-5p is associated with a deterioration in liver function and an increase in HCV severity. Validated target genes were obtained from the intersection between features selected based on the XGBoost model and predicted target genes based on miRNet. The results of hub detection indicated that inhibition or interference of SLC12A4 and CDK1 by drugs may have potential as an effective method of HCV therapy and progression prevention. Molecular docking revealed a strong affinity binding between paeoniflorin and diosmin with CDK1, which may result in a promising anti-HCV molecule.

## Figures and Tables

**Table 1:** The number of miRNAs and predicted target genes in Child–Pugh A and C, separately for up-regulated and down-regulated DEmiRNAs.

	Regulation	Predicted Target Genes	Key DEmiRNAs
Specific DEmiRNAs in Child–Pugh A	Down	3722	hsa-mir-152, hsa-miR-939, hsa-mir-195,
	Up	786	miR-27b
Specific DEmiRNAs in Child–Pugh C	Down	4724	hsa-mir-155, hsa-miR-99a,
	Up	240	hsa-miR-886-5p, hsa-miR-342-3p

**Table 2:** Comparison of prediction results of different machine learning models.

Model	PPV	Recall	F1-Score	Accuracy	AUC	BS	MCC
Random Forest	0.961	0.973	0.973	0.973	0.973	0.030	0.946
XGBoost	0.967	0.978	0.978	0.978	0.978	0.023	0.957
AdaBoost	0.956	0.973	0.971	0.972	0.973	0.130	0.946
Bagging	0.956	0.967	0.967	0.967	0.967	0.032	0.935
Boosting	0.956	0.967	0.967	0.967	0.967	0.030	0.935

PPV—positive predictive value; BS—Brier score; MCC—Matthews correlation coefficient.

**Table 3:** List of compounds, category, source, mechanisms, chemical formula, molecular weight, and binding affinity of 18 natural small molecule to CDK1.

Compound	Classifications	Source	Mechanism	Pubchem ID	Molecular Weight (g/mol)	Chemical Formula	Binding Energy/(kcal/mol)
Berberine	Minor alkaloid	*Coptis chinensis*	Inhibiting the AMPK pathway	2353	3.363.612	C20H18NO4	–4.57
Caffeine	Minor alkaloid	*Coffea*	Dampening the cAMP/PKA/CREB pathway	2519	1.941.906	C8H10N4O2	–5.27
Tetrandrine	Minor alkaloid	*Stephania tetrandra*	Inhibited TGF-β1-induced α-SMA secretion and collagen deposition	73,078	622.762	C38H42N2O6	–4.96
Capsaicin	Minor alkaloid	*Capsicum annuum* L.	Inhibiting the TGF-β1/Smad pathway via PPAR-γ activation	1,548,943	3.054.119	C18H27NO3	–3.44
Melatonin	Minor alkaloid	*Juglans regia*	Inhibiting TGF-β1/Smad Signaling Pathway	896	2.322.783	C13H16N2O2	–4.31
Oxymatrine	Minor alkaloid	*Sophora flavescens*	Modulation of TLR4-dependent inflammatory and TGF-β1 signaling inhibited NFkappaB transcriptional activity, TGF-β1 and α-SMA expression	114,850	264.369	C15H24N2O2	–5.39
Puerarin	Flavonoid monomer	*Puerariae lobata*	The regulation of NF-κB/IκBα, p38 MAPK, and Bcl-2/Bax signaling	5,281,807	4.163.781	C21H20O9	–5.51
Diosmin	Flavonoid monomer	*Bitter orange*	Inducing the expression of Nrf2 and its downstream antioxidant factors	5,281,613	6.085.447	C28H32O15	–6.01
Myricetin	Flavonoid monomer	*Myrica rubra*	Inhibits the activation of HSCs	5,281,672	3.182.351	C15H10O8	–4.22
Hesperetin	Flavonoid monomer	*Citrus reticulata*	Inhibiting TGF-β1/Smad pathway-mediated extracellular matrix progression and apoptosis	72,281	3.022.788	C16H14O6	–5.21
Icaritin	Flavonoid monomer	*Herba Epimedium*	Induce cell death in activated HSCs through mitochondria-mediated apoptosis	5,318,980	368.385	C21H20O6	–4.87
Naringenin	Flavonoid monomer	*Amacardium occidentale*	Blocking TGFβ-Smad3 and JNK-Smad3 pathways	932	2.722.528	C15H12O5	–5.41
Silibinin	Flavonoid monomer	*Silybum marianum*	Hepatoprotective, antioxidant, free radical scavenging, membrane stabilizing and anti-fibrotic activity	31,553	482.441	C25H22O10	–4.62
Artesunate	Monoterpene	*Artemisia*	Inhibition of LPS/TLR4/NF-κB signaling pathway	6,917,864	384.425	C19H28O8	–5.27
Betulinic acid	Monoterpene	*Betula platyphylla*	Modulating the TLR4/MyD88/NF-κB signaling pathway	64,971	456.711	C30H48O3	–5.82
Paeoniflorin	Monoterpene	*Paeonia lactiflora*	Regulating TGF-β1/Smads signaling pathway	442,534	480.466	C23H28O11	–6.32
Curcumin	Single phenol	*Curcuma longa*	Inhibition of the activation of HSCs and induction of their apoptosis	969,516	3.683.799	C21H20O6	–4.15
Resveratrol	Single phenol	*Veratrum nigrum*	Reduced collagen-1, TGF-β, NF-κB mRNA expression and desmin and α-SMA protein expression	445,154	2.282.433	C14H12O3	–4.75

**Table 4:** Characteristics of the subjects (mean ± SD).

Parameter/Disease	Control *n* = 22	HCV *n* = 42	Ch–P A *n* = 23	Ch–P B *n* = 11	Ch–P C *n* = 7
Sex (male/female)	11/9	30/28	16/14	11/10	3/4
Age (years)	63 ± 10	56 ± 7	57 ± 7	55 ± 8	52 ± 9
Total bilirubin (mg/dL)	0.59 ± 0.25	1.75 ± 1.26	1.03 ± 0.57	2.05 ± 0.84	3.62 ± 1.78
Albumin (g/dL)	3.89 ± 0.38	3.38 ± 0.57	3.67 ± 0.49	3.23 ± 0.45	2.71 ± 0.40
INR	1.14 ± 0.21	1.30 ± 0.28	1.20 ± 0.22	1.29 ± 0.17	1.71 ± 0.36

HCV—hepatitis C; Ch–P: A, B, C—Child–Pugh Class A, B, C; INR—International Normalized Ratio.

**Table 5:** (**a**) Confusion matrix; (**b**) Formula for evaluation metrics.

(**a**) **Confusion Matrix**, contains 4 different values (FP, TP, FN, TN) for evaluating the performance of classification models when the predictive scores are binary (represented as zeros and ones).			
		**Actual**	
		Positive (1)	Negative (0)
**Predicted**	Positive (1)	TP	TN
	Negative (0)	FP	FN
(**b**) **Evaluation metric** to assess the performance of the models using accuracy, precision (positive predictive value—PPV), recall (sensitivity), and F-Score (a harmonic mean of sensitivity).			
**Evaluation Metric**		**Formulation**	
Accuracy		$\;\frac{\mathrm{TP}\;+\;\mathrm{TN}}{N}$	
PPV		$\frac{\mathrm{TP}}{\mathrm{TP}\;+\;\mathrm{FP}}\;$	
Recall		$\frac{\mathrm{TP}}{\mathrm{TP}\;+\;\mathrm{FN}\;}$	
F-Score		$\frac{2\;*\;\mathrm{TP}}{2\;*\;\mathrm{TP}\;+\;\mathrm{FP}\;+\;\mathrm{FN}\;}$	

Abbreviations: TP, True Positive; TN, True Negative; N, total number of genes in the database; FP, False Positive; FN, False Negative; N, total number of samples.

## Data Availability

Not applicable.

## Supplementary Materials

The following supporting information can be downloaded at: https://www.mdpi.com/article/10.3390/ijms24087207/s1.
