# Supplementary material for: A Mixture Method for Robust Detection HCV Early Diagnosis Biomarker with ML Approach and Molecular Docking
Source: Int J Mol Sci. 2023 Apr 13;24(8):7207. doi: 10.3390/ijms24087207 (PMC10138470; doi:10.3390/ijms24087207)
Supplement: Supplementary file 1 [file ijms-24-07207-s001.zip › ijms-2316285-supplementary.pdf]

Supplementary file S1. Differentially expressed miRNAs

A. DEmiRNAs between total HCV patients and normal

miRNA_name	pvalupe	regulation	miRNA_name	pvalupe	regulation
hsa-miR-22	1.24E-08	down	hsa-miR-340#	3.46E-01	down
hsa-miR-92a	9.37E-09	down	hsa-miR-192#	4.17E-10	down
hsa-miR-99a	6.04E-07	down	hsa-miR-214#	0.000743	down
hsa-miR-125b	3.80E-08	down	hsa-miR-625#	8.44E-06	down
hsa-miR-148a	7.18E-01	down	hsa-miR-664	3.01E-07	down
hsa-miR-152	0.000908	down	hsa-miR-27b	1.71E-01	down
hsa-miR-192	1.20E-10	down	hsa-miR-30c	2.37E-11	down
hsa-miR-203	9.98E-06	down	hsa-miR-103	8.96E-12	down
hsa-miR-210	3.67E-08	down	hsa-miR-365	0.009989	down
hsa-miR-335	6.69E-07	down	hsa-miR-200a	3.35E-07	up
hsa-miR-30b	9.95E-16	down	hsa-miR-429	3.44E-09	up
hsa-miR-140	7.50E-07	down	hsa-miR-146b	2.34E-08	up
hsa-miR-660	1.49E-13	down	hsa-miR-886-5p	9.57E-06	up
hsa-miR-376c	5.13E-06	down	hsa-miR-200b	4.68E-08	up
hsa-miR-193a-5p	0.000405	down	hsa-miR-222	2.25E-13	up
hsa-miR-186	8.43E-10	down	hsa-miR-31	6.84E-07	up
hsa-miR-28-3p	3.41E-08	down	hsa-miR-939	5.64E-05	up

B. DEmiRNAs between Childpugh-A patients and normal

miRNA	P.Valupe	regulation	miRNA	P.Valupe	regulation
hsa-miR-27b	6.66E-16	down	hsa-miR-28	1.43E-07	down
hsa-miR-148a	6.29E-14	down	hsa-miR-340	1.78E-07	down
hsa-miR-30b	2.26E-13	down	hsa-miR-125b	3.56E-07	down
hsa-miR-660	2.14E-12	down	hsa-miR-140	4.72E-07	down
hsa-miR-103	1.28E-11	down	hsa-miR-376c	8.76E-07	down
hsa-miR-192	8.96E-11	down	hsa-miR-939	9.66E-07	up
hsa-miR-30c	8.14E-09	down	hsa-miR-200b	1.38E-06	up
hsa-miR-365	1.18E-08	down	hsa-miR-664	1.38E-06	down
hsa-miR-22	1.59E-08	down	hsa-miR-31	1.32E-06	up
hsa-miR-192#	1.66E-08	down	hsa-miR-200a	2.50E-06	up
hsa-miR-429	2.38E-08	up	hsa-miR-203	3.33E-06	down
hsa-miR-335	3.19E-08	down	hsa-miR-152	6.85E-06	down
hsa-miR-455-3p	4.98E-08	down	hsa-miR-195	7.27E-06	down
hsa-miR-186	1.10E-07	down	hsa-miR-222	1.32E-05	up

C. DEmiRNAs between Chilpugh-B patients and normal

miRNA	P.Valupe	regulation	miRNA	P.Valupe	regulation
hsa-miR-146b	9.55E-08	up	hsa-miR-365	6.54E-09	down
hsa-miR-192	1.94E-08	down	hsa-miR-429	1.16E-08	up
hsa-miR-30c	4.42E-08	down	hsa-miR-27b	2.48E-13	down
hsa-miR-222	3.07E-06	up	hsa-miR-92a	8.50E-06	down
hsa-miR-30b	7.81E-11	down	hsa-miR-192	2.25E-08	down
hsa-miR-148a	1.06E-12	down	hsa-miR-22	2.29E-06	down
hsa-miR-200b	5.86E-08	up	hsa-miR-660	2.01E-08	down
hsa-miR-186	1.23E-06	down	hsa-miR-103	1.17E-08	down
hsa-miR-455-3p	2.69E-07	down	hsa-miR-31	9.92E-07	up
hsa-miR-340	1.19E-06	down			

D. DEmiRNAs between Chilpugh-C patients and normal

miRNA	P.Valupe	regulation	miRNA	P.Valupe	regulation
hsa-miR-146b	3.45E-07	up	hsa-miR-92a	8.38E-08	down
hsa-miR-222	8.28E-15	up	hsa-miR-155	1.48E-05	down
hsa-miR-30c	4.20E-10	down	hsa-miR-27b	4.60E-12	down
hsa-miR-192	1.64E-05	down	hsa-miR-200a	3.43E-06	up
hsa-miR-30b	4.49E-13	down	hsa-miR-210	1.44E-09	up
hsa-miR-148a	1.76E-11	down	hsa-miR-22	6.34E-06	down
hsa-miR-342-3p	1.12E-05	up	hsa-miR-192	2.78E-07	down
hsa-miR-125b	5.92E-07	down	hsa-miR-660	7.60E-08	down
hsa-miR-186	1.64E-08	down	hsa-miR-193a-5p	2.35E-06	down
hsa-miR-99a	2.01E-07	down	hsa-miR-28	1.19E-06	down
hsa-miR-454	1.62E-05	down	hsa-miR-103	4.32E-07	down
hsa-miR-886-5p	6.58E-07	up	hsa-miR-340	1.09E-08	down
hsa-miR-664	3.06E-06	down	hsa-miR-625	1.97E-06	down
hsa-miR-365	3.20E-09	down	hsa-miR-455-3p	1.39E-05	down

Supplementary file S2. Common DEmiRNAs between child-A,B ,C and potential biomarkers for stage A and C

Common DEmiRNAs between child-A, B and C	DEmiRNAs only in child-A but not child- B or C	DEmiRNAs only in child-C but not child- A or B
hsa-miR-148a	hsa-miR-27b	hsa-miR-342-3p
hsa-miR-30b	hsa-miR-335	hsa-miR-99a
hsa-miR-660	hsa-miR-140	hsa-miR-454
hsa-miR-103	hsa-miR-376c	hsa-miR-886-5p
hsa-miR-192	hsa-miR-939	hsa-miR-155
hsa-miR-30c	hsa-miR-203	hsa-miR-210
hsa-miR-365	hsa-miR-152	hsa-miR-193a-5p
hsa-miR-22	hsa-miR-195	hsa-miR-625
hsa-miR-192		
hsa-miR-455-3p		
hsa-miR-186		
hsa-miR-340		
hsa-miR-222		

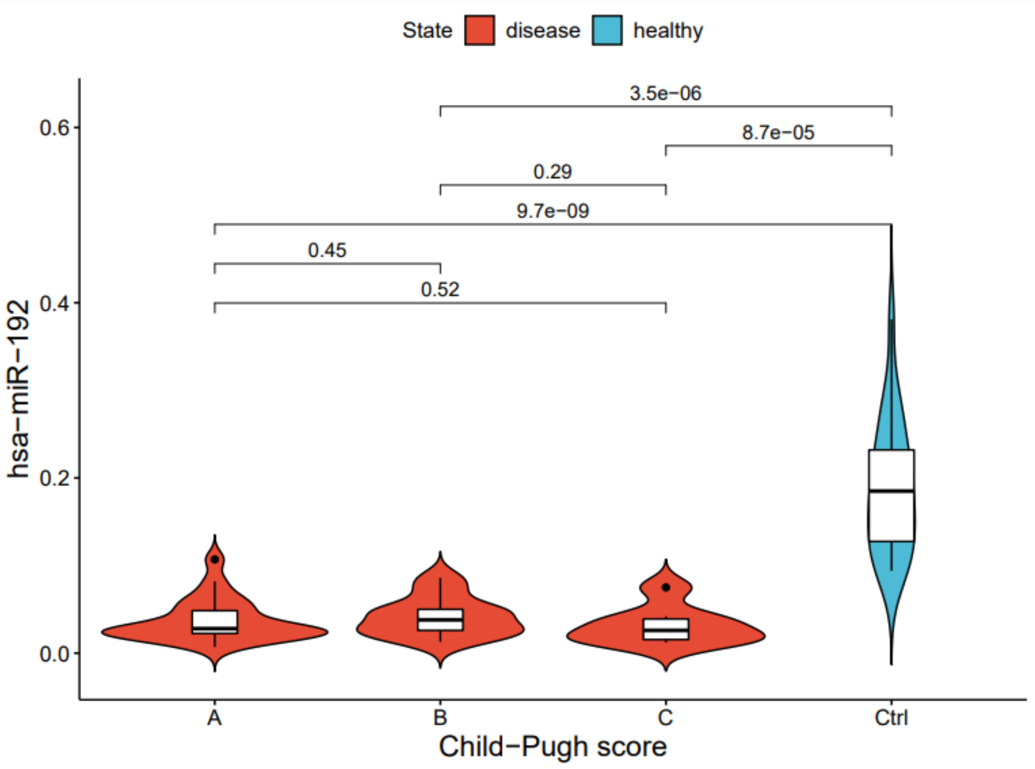

Supplementary file S3. Principal components analysis plot illustrated different pattern of 3 DEmiRNAs which shown significant increase in expression at functional stage C compare to healthy group, patients at stage A and B of HCV disease.

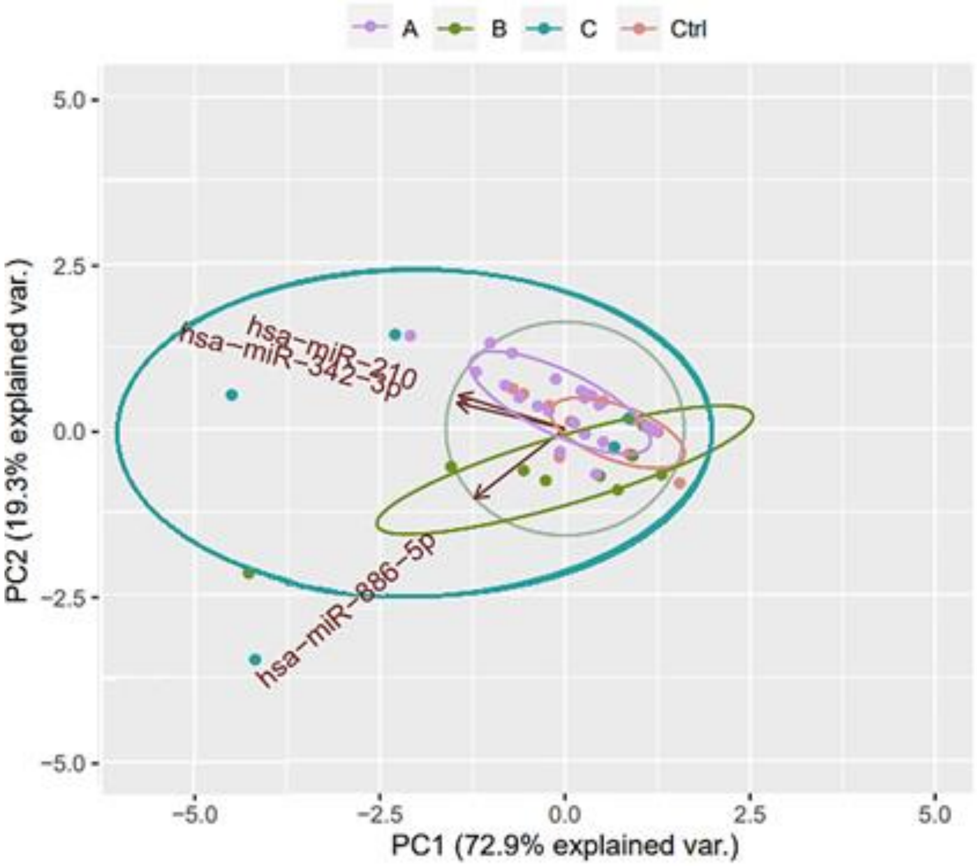

Supplementary file S4. A) The number of predicted target genes for specific downregulated and upregulated DEmiRNAs in Child-pugh A, target genes for specific downregulated and upregulated DEmiRNAs in Child-pugh C and the in related PPI network, **B)** predicted target genes for hsa-miR-342-3p

**A**	**DEmiRNAs**	**Number of targets**	**PPI network**
Downregulated DEmiRNAs in Child-pugh A	hsa-mir-140-5p	647	number of interactions in PPI network: 98926
	hsa-mir-152-3p	748	
	hsa-mir-195-5p	3538	
	hsa-miR-939	87	
Upregulated DEmiRNAs in Child-pugh A	hsa-miR-27b	858	*
Downregulated DEmiRNAs in Child-pugh C	hsa-mir-155-5p	5884	number of interactions in PPI network: 137326
Upregulated DEmiRNAs in Child-pugh C	hsa-miR-342-3p	240	*

B) predicted target genes for hsa-miR-342-3p

RFX3	GXYLT1	KCNA4	TASOR	UBE2D2	RRM2	DTD1	ETAA1	KCNB1
CDYL2	ENTPD6	FAM222B	FIGN	SLC35E3	SSBP3	ILDR2	ZNF20	FUT9
SPATA33	TSPAN4	MEOX2	FAXC	RCC2	HOXB8	ZBTB20	FOSB	IRAK4
SLC38A1	E2F3	CHRND	CLN3	LAMP5	NPAP1	KIAA0930	BCL2L10	JMJD1C
HTR2C	TGIF1	PLEKHB2	DHRSX	CASP2	TTBK2	RAD52	ENSA	KDM6B
ZFP28	EHF	ATG10	LATS2	SORL1	TIA1	RNF144B	ARHGEF28	ZNF225
PTGER4	RAD54L2	AUTS2	BTN2A1	JPH3	N4BP2L1	FRYL	PHF3	MLLT6
SLC6A8	PDE4D	AGPAT4	RAET1E	ACSL4	PLK2	GGNBP2	INO80D	MEX3A
PRPF40B	SLCO1A2	MED6	TMEM35A	ARID1B	GPBP1L1	UXS1	NEURL1B	ID4
KAT2A	SV2B	TMTC2	SLITRK4	GSTA4	DCUN1D1	OSBPL8	UCHL5	MRFAP1
APOLD1	ZNF519	NSG1	EDA	P2RY13	KLF6	SLC45A2	CNR1	CDKL5
UGCG	METTL21A	SLC30A9	DAZAP1	LACC1	B3GNT7	CHST15	MYLK2	FAM126B
ADI1	COG2	RPS6KA5	ZNRF3	DNAJC28	CLEC7A	FBXO9	DRAM1	PDE12
KDM4D	GPR143	MAN2A1	MAF	SEMA4B	OR4N4	FAM222A	NEO1	ZNF652
EPC1	EEA1	ATXN7	DTNBP1	MAP3K7CL	TXK	NTM	ZNF189	ZNF84
FBLIM1	CCSAP	SCN1B	CCDC141	CCDC28A	SNAP25	L3MBTL2	SLC43A2	PPP3R1
LPCAT1	CMIP	ATXN2L	ISL2	MAP3K20	KIAA0825	BMP7	AGPS	SLC17A8
CAMK2D	RIC8B	ZBTB5	POM121	IQCE	PTER	DDX59	SPCS1	WDR20
ZNF740	JADE1	ZNF568	S100A7A	CACNA1C	CTNND2	PDGFRA	KCTD16	FAM192A
EP300	ZNF117	PAK2	NSD1	ADGRG2	NABP2	NBEA	FKBP14	KLHL28
MMS19	FUT8	SLC6A19	NRARP	MAGEB2	SOX21	ARHGEF9	USP13	SH3TC2
TANC2	COL1A2	CLCN6	OSER1	SOX6	ZNF322	MMS22L	PITPNM2	IBTK
ATXN1L	ZBTB4	LIN28B	UBN1	ZBTB40	C5orf24	BIRC6	WBP1L	RICTOR
TLE1	ZNF329	ELAVL2	CUX2	ZEB1	FASTKD2	ANKDD1A	ASPH	CA12
NDEL1	TXNDC15	SLC4A8	RWDD1	MBNL3	SAMD4A	ITGAM	FXYD3	RGS4
TRIM56	GJA1	DCAF12	SGMS2	IER3	P4HA2	NUFIP2	NUBPL	ANKRD49

Supplementary file S5. The least number of features were that obtained through recursive feature elimination based on XGBoost (only 279 of total (1880)shown here)

9.98128999672773e-05 : NM_005033	5.165564563330471e-05 : NM_053282	5.887449964242272e-05 : NM_000235
9.978691741061648e-05 : AK000832	5.164806097079472e-05 : NM_172178	5.87965152915871e-06 : NM_207123
9.96226723237465e-05 : NM_001281	5.161245368192894e-05 : NM_025108	5.802209418065859e-06 : NM_020665
9.936087339836583e-05 : NM_213568	5.161245368192894e-05 : NM_021188	5.783692752332764e-06 : NM_020141
9.917723116680856e-05 : NM_006585	5.152667628948463e-05 : NM_032442	5.745503266637324e-06 : NM_002763
9.906261271208928e-05 : NM_033312	5.151282875487311e-05 : NM_005551	5.712772876334222e-05 : NM_004451
9.898749204641053e-05 : NM_002906	5.148575437708041e-05 : NM_018044	5.6842007175137724e-05 : NM_134470
9.89283473673878e-05 : NM_030917	5.146152578678531e-05 : NM_021964	5.6831514555944096e-05 : NM_021126
9.862644560050903e-05 : NM_001001	5.1455612714991063e-05 : NM_015414	5.681492836977399e-05 : NM_203430
9.853335215103902e-05 : D86975	5.1450742240215915e-05 : NM_006759	5.649649323207042e-06 : NM_000221
9.843413817834092e-05 : NM_145691	5.1444233607421484e-05 : NM_001961	5.548191697654563e-05 : NM_022350

9.838709677419359e-05 : THC2158597	5.138294048938273e-05 : NM_014675	5.512173018146929e-05 : NM_021103
9.838709677419358e-05 : NM_016227	5.1382940489382726e-05 : NM_177441	5.50088607341208e-06 : NM_006770
9.8372405554726e-05 : NM_000936	5.126778030121921e-05 : AW901755	5.488079172289698e-05 : NM_018234
9.802253478525383e-07 : NM_013446	5.125717396321729e-05 : AL110257	5.479246977806056e-05 : NM_006252
9.797268039207965e-05 : NM_178348	5.1253995757774906e-05 : NM_004541	5.4761378244e-05 : ENST00000361624
9.778394466395994e-05 : NM_003001	5.1248254385506354e-05 : BE779590	5.47357926233174e-05 : NM_002413
9.776008213449521e-05 : NM_012184	5.120402916950843e-05 : NM_014372	5.47281888377936e-05 : NM_152771
9.775351925238071e-05 : NM_019892	5.113963845952319e-05 : NM_015294	5.470361402564792e-05 : NM_024308
9.762853659120541e-05 : NM_207113	5.111669330204566e-05 : NM_005021	5.470361402564792e-05 : NM_014303
9.760723039547834e-06 : NM_133455	5.108763114012458e-05 : AK027463	5.468617898629094e-05 : NM_152478
9.747137001352267e-05 : NM_130809	5.1080069864928606e-05 : NM_004933	5.4664804677873444e-05 : NM_017453
9.721890190453421e-05 : NM_005143	5.1062185757749745e-05 : NM_003426	5.4651336419292213e-05 : NM_006276
9.714705187065306e-05 : NM_022074	5.101553166069296e-05 : AF159055	5.464644051600573e-05 : NM_006767
9.684157179202852e-05 : U43747	5.1011341661004545e-05 : NM_014893	5.4633871762422805e-05 : NM_015331
9.669719989003742e-05 : NM_006249	5.1005248344494475e-05 : AK091801	5.458417705680393e-05 : NM_001668
9.669403967307288e-06 : NM_002493	5.095102117830898e-05 : NM_005268	5.452410888025301e-05 : NM_006715
9.66658215010142e-05 : NM_018485	5.089139928916657e-05 : NM_147150	5.4504755772401705e-05 : AF071111
9.643849476900211e-05 : NM_000108	5.087872193677054e-05 : NM_001127	5.448636430318096e-05 : NM_002799
9.62735018012446e-05 : NM_001124	5.0813516292009175e-05 : NM_144575	5.445637973581064e-05 : NM_003072
9.578930838773358e-05 : NR_001543	5.07874835218206e-05 : NM_005202	5.445564226926077e-05 : NM_003869
9.563983873376138e-05 : NM_003222	5.0779515726921905e-05 : NM_015193	5.443944859505411e-05 : NM_022355
9.563983873376138e-05 : BE073457	5.075524833589351e-05 : NM_018117	5.442184999041636e-05 : NM_002296
9.549399023083237e-05 : NM_006213	5.074766953219993e-05 : NM_021135	5.435592378999874e-05 : NM_002782
9.492866320463429e-05 : NM_152889	5.074766953219993e-05 : NM_016585	5.43098384137314e-05 : NM_004805
9.485645933014356e-05 : NM_015578	5.074312333629104e-05 : NM_000847	5.425411839790716e-05 : NM_014743
9.466177415420814e-05 : NM_199168	5.059243371262299e-05 : NM_002611	5.424085342949443e-05 : NM_003472
9.428426396146959e-05 : BC038245	5.058487541857831e-05 : NM_004653	5.421449935906551e-05 : NM_032329
9.415194649288288e-05 : NM_080879	5.058185441149871e-05 : NM_080862	5.418528884736561e-05 : BC043009
9.415194649288288e-05 : NM_017893	5.055873132685925e-05 : NM_016023	5.418189275153154e-05 : AF264626
9.395737689487128e-05 : NM_017566	5.053794373166099e-05 : NM_018389	5.417982174432275e-05 : NM_006136
9.387935263431834e-05 : NM_181671	5.052622860012319e-05 : NM_019103	5.4163378058405595e-05 : NM_145740
9.383442454308594e-05 : NM_175063	5.0486965867845296e-05 : NM_005864	5.415212238741661e-05 : AB023177
9.376150804028414e-05 : R79606	5.047904521966198e-05 : NM_000195	5.415098317195198e-05 : NM_014248
9.37467982650866e-05 : NM_032933	5.0447387463962024e-05 : NM_003536	5.4138931558286553e-05 : NM_004058
9.37467982650866e-05 : NM_024911	5.034722222222222e-05 : NM_002410	5.413120281832576e-05 : NM_173522
9.374679826508657e-05 : NM_006863	5.0366473927e-05 : ENST00000219837	5.412963248345862e-05 : NM_013240
9.370199692780341e-05 : NM_005333	5.030823238319456e-05 : THC2037524	5.4121862915926315e-05 : NM_014602
9.368310920417351e-05 : NM_052857	5.0261176511736426e-05 : NM_138476	5.4118156631094826e-05 : NM_000523
9.327190622631626e-06 : NM_005809	5.0226430629888854e-05 : NM_001166	5.410417492796269e-05 : NM_001621
9.294487025579463e-05 : D00022	5.017535201262236e-05 : NM_004314	5.410300674206188e-05 : NM_014902
9.265289684137851e-05 : AI310734	5.012138188608777e-05 : NM_015079	5.4101099357511126e-05 : NM_152314

9.265257433747586e-05 : NM_019598	5.0109529025191686e-05 : NM_031905	5.405311319664984e-05 : NM_007192
9.255943742438306e-05 : NM_003474	5.010940428805066e-05 : NM_018066	5.40510367077567e-05 : NM_019009
9.255943742438306e-05 : BC040697	5.0104540023894864e-05 : BC062428	5.404813776281364e-05 : NM_022786
9.238286356837753e-07 : NM_153813	5.00211131765e-05 : ENST00000273375	5.401910161655016e-05 : NM_018940
9.138712680474716e-05 : AF516696	4.9825881686674176e-05 : NM_024296	5.4017682561605104e-05 : NM_144984
9.137550088005093e-05 : NM_006805	4.97845051848658e-05 : NM_007372	5.401369315927367e-05 : NM_203462
9.134744975890262e-05 : THC2114831	4.9760679681939526e-05 : NM_018445	5.3999446726980165e-05 : NM_153461
9.132078296343427e-05 : NM_004497	4.971421120118228e-05 : NM_201523	5.3986840572697196e-05 : NM_024329
9.129549808429119e-05 : NM_170744	4.971421120118228e-05 : NM_014109	5.3926200668331275e-05 : NM_006037
9.124906507105462e-05 : NM_000065	4.970715558950853e-05 : NM_001762	5.392387199499911e-05 : NM_021795
9.110800779645576e-05 : NM_178545	4.967858228727794e-05 : NM_014415	5.3922133825281534e-05 : NM_005635
9.110800779645576e-05 : NM_024952	4.967746970658397e-05 : NM_024585	5.391684198223444e-05 : NM_025027
9.109916367980886e-05 : NM_005195	4.967746970658397e-05 : AK094686	5.390816145385303e-05 : NM_018342
9.109168153750811e-05 : NM_002461	4.95259490959138e-06 : NM_000592	5.390674838561401e-05 : NM_005762
9.109168153750811e-05 : AF090921	4.946488357235172e-05 : BC013629	5.390033851055363e-05 : NM_183013
9.094235687318957e-05 : NM_006295	4.931322280025452e-05 : NM_016091	5.389591157635903e-05 : NM_015192
9.024426421043912e-05 : THC2094604	4.922479172114919e-05 : NM_001824	5.3893794030404294e-05 : NM_178231
8.961210933275843e-05 : NM_005224	4.919832421008612e-05 : NM_013237	5.3891165077078915e-05 : NM_016298
8.959697327005343e-05 : NM_147223	4.9198324210086125e-05 : NM_139280	5.3871981848048286e-05 : NM_147130
8.945149856379292e-05 : NM_024108	4.919354838709678e-05 : NM_015046	5.3869428853818345e-05 : NM_022361
8.945149856379292e-05 : NM_006347	4.899598393574297e-05 : AK000809	5.3866881747162124e-05 : NM_015074
8.944281524926686e-05 : NM_032193	4.873360046936048e-05 : NM_012255	5.386413364730653e-05 : NM_012252
8.939569938948292e-06 : NM_152734	4.871863987141473e-05 : NM_144675	5.385194310577092e-05 : NM_018677
8.934462700039983e-06 : NM_032283	4.8718639871414734e-05 : AF230201	5.3844314900972826e-05 : NM_020445
8.932287440621845e-05 : AL133112	4.8704417580498276e-05 : NM_005615	5.382322594176891e-05 : NM_014300
8.886099391346896e-06 : NM_007162	4.869093231162196e-05 : NM_024529	5.381054662822621e-05 : AK000820
8.885265369414897e-06 : NM_152435	4.86909162196e-05 : ENST00000273176	5.381024005402123e-05 : NM_032710
8.861079764354832e-05 : AK055261	4.8666168037895784e-05 : NM_006589	5.3804938721131706e-05 : NM_006361
8.787515006002399e-05 : NM_014290	4.8666168037895784e-05 : NM_005235	5.380482813184424e-05 : NM_000876
8.74636874845975e-05 : NM_153614	4.865488680011864e-05 : NM_012102	5.379758658049084e-05 : NM_014594
8.746368748459752e-05 : NM_005264	4.8654886800118634e-05 : NM_153228	5.3764295662340634e-05 : NM_000786
8.745519713261646e-05 : NM_153609	4.858622062923137e-05 : NM_025237	5.37554128714349e-05 : NM_004869
8.745519713261646e-05 : NM_005906	4.8582230153337655e-05 : NM_015020	5.373466542659561e-05 : X84195
8.74375685905619e-05 : NM_018998	4.8582230153337655e-05 : NM_000302	5.373379397582514e-05 : NM_003895
8.733316005105792e-05 : NM_005253	4.820610083767979e-05 : NM_147202	5.373293648916453e-05 : NM_014716
8.682965019038845e-06 : NM_175622	4.809898290866792e-06 : AK131226	5.371753064807949e-05 : NM_006303
8.658146631703498e-06 : NM_024054	4.8041749252e-06 : ENST00000341954	5.3715291259564044e-05 : AK128645
8.636971358649798e-05 : NM_178428	4.798674202362299e-06 : NM_001671	5.36899296542122e-05 : NM_016495
8.594625968227558e-05 : NM_139156	4.795741112342389e-05 : NM_178532	5.368973509946787e-05 : NM_144718
8.531127627267642e-05 : NM_014172	4.7943411055802994e-05 : NM_033044	5.368343573988737e-05 : NM_021815
8.52576197749758e-05 : BF758462	4.7943411055802994e-05 : NM_032943	5.3665317979043414e-05 : NM_007241

Supplementary file S6. A) Up and down regulated target genes for DEmiRNAs in Child-pugh A and C**.**

A) Up-regulated target genes for DEmiRNAs in Child-pugh A,

ACTR5	CDK2	FGFRL1	KATNBL1	NDUFA2	RALA	SPCS3	ZCCHC2	CDKN1B
ADA	MYC	FHL1	KLK10	NISCH	RB1CC1	SPEN	ZDHHC21	FGF2
ADAM9	CUL3	FKBP4	KMT2C	NSUN2	RC3H2	SPRED1	ZFYVE16	IGF2BP1
AIDA	YWHAQ	FXR1	KMT2E	NUCKS1	REV3L	STAT1	ZNF426	MYO6
ALDH1A1	EGR1	SMAD4	LAMC1	OSTM1	RIOK3	STK24	ZNF652	RAB10
ARID1B	HSP90AA1	ZEB1	LARP1	PANK3	RNF149	STRADB	ZNF800	SOX4
ARIH1	SRSF1	SMAD2	LDLR	PAX6	RNF19A	TFRC	GSK3B	YTHDC2
BAZ1B	RB1	ITGA5	LIN28B	PDGFRA	RPRD2	TGFBR1	CDC6	CNKSR2
BCL2L11	FN1	BAP1	LYRM2	PHACTR2	RPUSD2	TM7SF3	SUCO	FEN1
SLC12A4	SMAD4	GNAI3	MAP3K14	FOS	SEC61A1	TMED4	FGF9	IGF1R
BRAP	EFHD2	GPR107	MCM10	PLEKHA8	SIRPA	TSC22D2	IGFBP5	MVP
BTBD1	EIF4B	HCCS	GTF2A1	POLDIP2	SMCR8	TSPAN12	MYO9A	PUM2
CAMK2N1	ENPP4	HDAC4	SOCS3	POMT2	SMURF1	UPF1	RAB30	SOX2
CAPN1	EP300	HDAC7	ILK	CALR	SNORD12C	VCL	SOX9	YOD1
CASD1	ESR2	HMGN5	BMI1	PPP1R12A	SNX11	VEGFA	ZC3H11A	SOAT1
CEBPD	EVI5	HOXD12	MELK	PRDM1	SNX16	VEZF1	PRPF8	YIPF4
CLK1	FBXW11	IFNLR1	MSL3					

B) Down-regulated target genes for DEmiRNAs in Child-pugh A

BARHL1	FBLN5	KRT3	PCDHGA12	CDR2L	ILK	CDKN1A	GTF2A1	BAP1
BCAS1	FIS1	KRTAP10	PDZD7	ERAL1	BMI1	HS6ST1	KIF1A	KDELR1
BTBD9	FLJ44635	LNX2	PEAR1	FAM193B	MELK	NOVA2	PADI2	PA2G4
C20orf203	FOXP4	LOXL3	PFN1	IFT140	FOS	SOCS3	CAVIN1	NOS2
C22orf46	GRIK3	LRRC25	PIRT	IQSEC3	CALR	SMAD40	HECTD3	
C3orf18	GRM4	LSAMP	POLE3	KCNK5	OLFML2A	HLA-DRB1	NGFR	

C) Down-regulated target genes for DEmiRNAs in Child-pugh C

MAP3K7CL	EGR1	ZNF740	AUTS2	BTN2A1	JPH3	LATS2	SORL1	RAD54L2
ATXN7	HSP90AA1	JADE1	CDKL5	N4BP2L1	MMS19	FUT8	DHRSX	CASP2
DTNBP1	KDM6B	ZNF568	FAM126B	NRARP	MYC	CUL3	YWHAQ	MRFAP1
SOX21	ZNF225	S100A7A	PDE12	CTNND2	MEX3A	ID4	SLC6A19	TTBK2
CACNA1C								

D) Up-regulated target genes for DEmiRNAs in Child-pugh C

RNF2	CALU	CFL2	DAG1	ENTPD1	GLB1	ITGB5	MGST2	MYO6
RORA	SELE	RPL39	DBN1	EPB41L2	GLG1	ITK	MIA2	NARS
ADH5	SRSF1	CLTC	DDB2	EPRS	GNAS	JARID2	MITF	NASP
AGL	SRSF2	COL4A2	DDX10	ESRRA	GPM6B	JCHAIN	MKLN1	NEU1
AGTR1	CBFB	CPD	DHCR24	ETS1	GSK3B	JUN	MLH1	NEUROG1

AKT1	CBL	CPT1A	DMD	EZH1	HAL	JUNB	MMP16	NFKB1
S100A11	CCND1	CRAT	DNAJB1	F5	HIF1A	JUP	MOV10	NFYC
SCD	YWHAQ	CS	DNMT1	FADS1	HIVEP2	KCNN3	MPP2	NKX3-1
CCL2	EGR1	CSE1L	DOCK1	FDFT1	HK2	KIF22	MSH2	NOS3
SDCBP	HSP90AA1	CSF1R	DR1	FGF2	HLA-DPA1	KLF9	MSH6	NOTCH2
AP1G1	SRSF1	CSNK1A1	DSG2	FGF7	HMGCS1	KPNA5	MTAP	NOVA1
APAF1	RB1	CSNK1G2	E2F2	FKBP3	ICAM1	KRAS	MUT	NR3C1
APC	FN1	CSRP2	ECI1	FLI1	IFNGR1	KRT6B	MXI1	NT5E
SLC1A5	SMAD4	CTLA4	EDN1	FLNA	IGF2R	LPL	MYB	OLR1
ASPH	ZEB1	CTNNA1	EEF1A2	FLNB	IL13RA1	MAP3K10	MYBL1	PHC2
ATP6V1C1	SMAD2	CTNNB1	EEF2	FLT1	IL2	MAPK14	MYC	RBPJ
AXL	ITGA5	CUX1	EGFR	FOS	IL6	MARCKS	MYD88	RHOA
B4GALT1	CDK5	CXCL8	EIF3E	FOXE1	INPP5A	MCAM	MYLK	RUNX2
BACH1	CDKN1B	CYP1A1	EIF4A1	FOXO3	INPP5D	MECP2	MYO10	S1PR1
BCAT1	CDKN2A	CYP51A1	EIF4G2	GALC	ITGB4	MEF2A	MYO1D	SLC11A2
BCL6	CEBPB	CYR61	ELK4	GATM	GCLC	MEIS1	MYO1E	SMAD1
SMAD2	SMAD3	SMAD4	SMAD5	TACSTD2	MEST			

Supplementary file S7. A) PPI network information for up and down regulated target genes of DEmiRNAs in Child-pugh A and C,

A) PPI for up-regulated target genes of DEmiRNAs in Child-pugh A

FKBP4	FXR1	SMAD2	ARIH1	FOS	BAP1	PDGFRA	SOX9	ACTR5
RALA	REV3L	KMT2C	ZDHHC21	KMT2E	MCM10	TGFBR1	RC3H2	EVI5
RB1CC1	TFRC	EP300	YWHAQ	BID	ADAM9	SPRED1	ALDH1A1	IGF2BP1
HDAC7	STAT1	CUL3	FGF9	CALR	SPCS3	BRAP	CLK1	EIF4B
YTHDC2	ZEB1	FGF2	BCL2L11	RPUSD2	CAPN1	LIN28B	ESR2	NISCH
CDC6	SMURF1	HDAC4	FHL1	SOX2	GTF2A1	LAMC1	BTBD1	MAP3K14
VCL	SOAT1	NSUN2	MVP	GSK3B	LDLR	SPEN	SOX4	FN1
TSPAN12	SUCO	RAB10	ILK	IFNLR1	PRPF8	FEN1	SOCS3	MYO9A
CDKN1B	RPRD2	FBXW11	FGFRL1	HSP90AA1	MELK	PAX6	ITGA5	MYC
IGFBP5	PRDM1	CDK2	SIRPA	LARP1	UPF1	ARID1B	PPP1R12A	CNKSR2
EGR1	GNAI3	RB1	SMCR8	ZFYVE16	VEGFA	BMI1	BAZ1B	SRSF1
SEC61A1	MYO6	SMAD4	CEBPD	PUM2				

Number of interactions: 625

B) PPI for down-regulated target genes of DEmiRNAs in Child-pugh A

LOXL3	FOS	CALR	NOS2	KDELR1	FBLN5	SOCS3	BMI1	ILK	CDKN1A	BAP1
**Number of interactions:** 22										

C) PPI for up-regulated target genes of DEmiRNAs in Child-pugh C

CYP51A1	DR1	DNAJB1	ATP6V1C1	MCAM	FLNB	MITF	FADS1	MYLK
ITGB4	SCD	JCHAIN	GATM	FGF2	MYO10	ITGB5	HMGCS1	COL4A2
GLG1	GNAS	NASP	BCL6	ICAM1	CSNK1A1	ADH5	MYO1D	ZEB1
FKBP3	ENTPD1	CSNK1G2	ESRRA	CPT1A	SMAD1	NOS3	GSK3B	DMD
EEF1A2	JUN	KRAS	IL6	RB1	MYBL1	CFL2	MXI1	E2F2
EIF3E	DHCR24	NOTCH2	FOXO3	FGF7	FLI1	AXL	SELE	RPL39
MEST	RUNX2	NARS1	HLA-DPA1	MEIS1	EIF4G2	ECI1	SMAD3	EPRS1
CPD	IL13RA1	DDB2	MECP2	MMUT	HIF1A	JUNB	HSP90AA1	RNF2
CCL2	NEU1	PHC2	NFYC	EGFR	NOVA1	CTNNA1	MYB	HIVEP2
NFKB1	KLF9	APC	ITK	DOCK1	SMAD5	S1PR1	JARID2	IFNGR1
IL2	EDN1	NT5E	CCN1	FLT1	DAG1	CEBPB	CUX1	NEUROG1
CCND1	ASPH	SRSF1	MOV10	SMAD4	SLC1A5	FOS	CTNNB1	KPNA5
CDKN1B	CYP1A1	SDCBP	RHOA	CSF1R	APAF1	CXCL8	RBPJ	INPP5A
MAPK14	B4GALT1	HAL	MYD88	MMP16	AKT1	GLB1	MEF2A	FLNA
NR3C1	NKX3-1	GALC	EZH1	MYO1E	CLTC	EEF2	FN1	MYO6
MLH1	YWHAQ	RORA	INPP5D	HK2	MYC	OLR1	IGF2R	CBL
MSH2	SRSF2	DSG2	CBFB	DBN1	FDFT1	DDX10	ELK4	CDK5
MSH6	ETS1	SMAD2	CDKN2A	ITGA5	MGST2	CRAT	DNMT1	AGL
EGR1	AP1G1	CSE1L	AGTR1	EIF4A1	MAP3K10	JUP		
**Number of interactions:** 2034								

D) PPI for down-regulated target genes of DEmiRNAs in Child-pugh C

EGR1	KDM6B	CUL3	TTBK2	JPH3	HSP90AA1	YWHAQ	LATS2	ATXN7	MYC
**Number of interactions:** 16									

Supplementary file S8. Statistical features of networks for validated target gens of DEmiRNAs in functional state A and C.

Network name	Nodes	Edges	Avg. neighbors	diameter	Path length	coefficient

Target genes of down-regulated DEmiRNAs in Child-pugh A	104	626	6.11	9	3.07	0.27
Target genes of up-regulated DEmiRNAs in Child-pugh A	11	22	2.57	3	1.76	0.4
Target genes of down-regulated DEmiRNAs in Child-pugh C	172	2034	11.82	6	2.69	0.45
Target genes of up-regulated DEmiRNAs in Child-pugh C	10	16	1.71	3	2	0.01

Supplementary file S9. The modules of networks for each group using Cytoscape (MCODE plug-in)

A. Parameters-up-regulated target genes of DEmiRNAs in Child-pugh A

Network Scoring:
Include Loops: false Degree Cutoff: 2
Cluster Finding:
Node Score Cutoff: 0.2 Haircut: true Fluff: false K-Core: 2 Max. Depth from Seed: 100

Cluster	Score (Density*#Nodes)	Nodes	Edges	Node IDs
1	11.429	27	160	FN1, FOS, CDK1, SOX2, FGF2, SMAD4, SOX9, TGFBR1, MYC, GSK3B, STAT1, SOCS3, CDKN1B, CDK2, CUL3, SLC12A4, EGR1, SRSF1, RB1, SMAD4, ZEB1, ITGA5, BAP1, GTF2A1, ILK, BMI1, MELK, CALR
2	3.333	4	10	CDC6, CDK2, BCL2L11, CDKN1B

B. Parameters-up-regulated target genes of DEmiRNAs in Child-pugh C

Network Scoring:
Include Loops: false Degree Cutoff: 2
Cluster Finding:
Node Score Cutoff: 0.2 Haircut: true Fluff: false K-Core: 2 Max. Depth from Seed: 100

Cluster	Score (Density*#Nodes)	Nodes	Edges	Node IDs
1	18.1	21	362	MAPK14, SMAD4, JUN, CCND1, EGFR, CTNNB1, EGR1, FOXO3, FN1, RHOA, FOS, SMAD2, AKT1, MYC, CEBPB, KRAS, CDKN2A, SMAD3, IL6, FGF2
2	6.375	17	102	NR3C1, SELE, DNMT1, RB1, BCL6, ICAM1, GSK3B, IL2, RUNX2, CCL2, HIF1A, EDN1, NOS3, CXCL8, ETS1, ZEB1, FLT1
3	5.6	6	28	SCD, FADS1, HMGCS1, CYP51A1, DHCR24, FDFT1
4	4	4	12	FLNB, ITGB5, ITGA5, ITGB4
5	3	3	6	CPD, IGF2R, AP1G1
6	3	3	6	EPRS1, EIF4G2, EIF3E
7	3	3	6	NEU1, GLB1, GALC
